# Supplementary material for: From lowlands to highlands: how elevation and habitat complexity drive anuran multidimensional diversity?
Source: PeerJ. 2025 Oct 8;13:e19561. doi: 10.7717/peerj.19561 (PMC12514998; doi:10.7717/peerj.19561)
Supplement: Supplemental Information 1 — Size = maximum body size (snout-vent length) in mm; habitat = habitat preference (F = forest, A = open area); call = calling site (ff = forest floor, len = lentic waters, lot = lotic waters, sh = shrubs, br = bromeliads; can = canopy); r_mode = reproductive mode (following Nunes-de-Almeida et al., 2022), hab = habit (Ar = arboreal, Cr = cryptozoic, Se = semi-arboreal, Phy = phytotelmata, Te = terrestrial); and act = activity period (D = diurnal and N = nocturnal). [file peerj-13-19561-s001.docx]

**Appendix.** List of species studied in alphabetical order with their functional traits. Size = maximum body size (snout-vent length) in mm; habitat = habitat preference (F = forest, A = open area); call = calling site (ff = forest floor, len = lentic waters, lot = lotic waters, sh= shrubs, br = bromeliads; can = canopy); r_mode = reproductive mode (following *Nunes-de-Almeida et al., 2022*), hab = habit (Ar = arboreal, Cr = cryptozoic, Se = semi-arboreal, Phy = phytotelmata, Te = terrestrial); and act = activity period (D = diurnal and N = nocturnal)

| **Species** | **Size** | **Habitat** | **Call** | **R_mode** | **Hab** | **Act** |
| --- | --- | --- | --- | --- | --- | --- |
| *Adelophryne* sp. | 14 | F | ff | m27 | Cr | N |
| *Aplastodiscus ibirapitanga* | 41 | F | lot | m10 | Ar | N |
| *Aplastodiscus weygoldti* | 38 | F | lot | m10_m16 | Ar | N |
| *Bahius bilineatus* | 26 | F | ff | m27 | Cr | D_N |
| *Bokermannohyla circumdata* | 54 | F | lot | m12 | Ar | N |
| *Bokermannohyla lucianae* | 51 | F | br | m16 | Ar | N |
| *Brachycephalus pulex* | 8 | F | ff | m27 | Cr | N |
| *Chiasmocleis crucis* | 20 | F | len | m1 | Cr | N |
| *Dendropsophus anceps* | 42 | F_A | len | m1 | Ar | N |
| *Dendropsophus novaisi* | 34 | A | len | m1 | Ar | N |
| *Gastrotheca pulchra* | 33 | F | can | m69 | Ar | N |
| *Haddadus binotatus* | 57 | F | ff | m27 | Te | N |
| *Boana crepitans* | 62 | A | len | m1_m12 | Ar | N |
| *Boana faber* | 89 | F_A | len | m1_m12 | Ar | N |
| *Boana pombali* | 57 | F | len_lot | m16 | Ar | N |
| *Ischnocnema* gr*. parva* | 20 | F | ff | m27 | Cr | N |
| *Ischnocnema verrucosa* | 24 | F | ff | m27 | Cr | N |
| *Phasmahyla spectabilis* | 46 | F | lot | m37 | Ar | N |
| *Phyllodytes maculosus* | 49 | F | br | m4 | Phy | N |
| *Phyllodytes magnus* | 41 | F | br | m4 | Phy | N |
| *Phyllodytes megatympanum* | 22 | F | br | m4 | Phy | N |
| *Phyllodytes melanomystax* | 23 | F | br | m4 | Phy | N |
| *Phyllodytes* sp. | 26 | F | br | m4 | Phy | N |
| *Phyllodytes wuchereri* | 27 | F | br | m4 | Phy | N |
| *Phyllomedusa burmeisteri* | 60 | F_A | len | m37 | Ar | N |
| *Physalaemus erikae* | 26 | F_A | len | m45 | Te | N |
| *Pristimantis* sp. 2 | 31 | F | sh | m27 | Se | N |
| *Pristimantis* sp. 1 | 33 | F | br_sh | m27 | Ar | N |
| *Pristimantis vinhai* | 25 | F | sh | m27 | Se | N |
| *Proceratophrys schirchi* | 50 | F | Lot | m16 | Te | N |
| *Rhinella crucifer* | 101 | F_A | len_lot | m2_m16 | Te | N |
| *Rhinella hoogmoedi* | 56 | F | lot | m16 | Te | N |
| *Scinax eurydice* | 53 | F_A | len | m1 | Ar | N |
| *Ololygon strigilata* | 38 | F | lot | m16 | Ar | N |
| *Trachycephalus mesophaeus* | 69 | F | len | m1 | Ar | D_N |
| *Trachycephalus nigromaculatus* | 92 | F | len_lot | m16 | Ar | N |
