# Supplementary figures and images for: From lowlands to highlands: how elevation and habitat complexity drive anuran multidimensional diversity?
Source: PeerJ. 2025 Oct 8;13:e19561. doi: 10.7717/peerj.19561 (PMC12514998; doi:10.7717/peerj.19561)

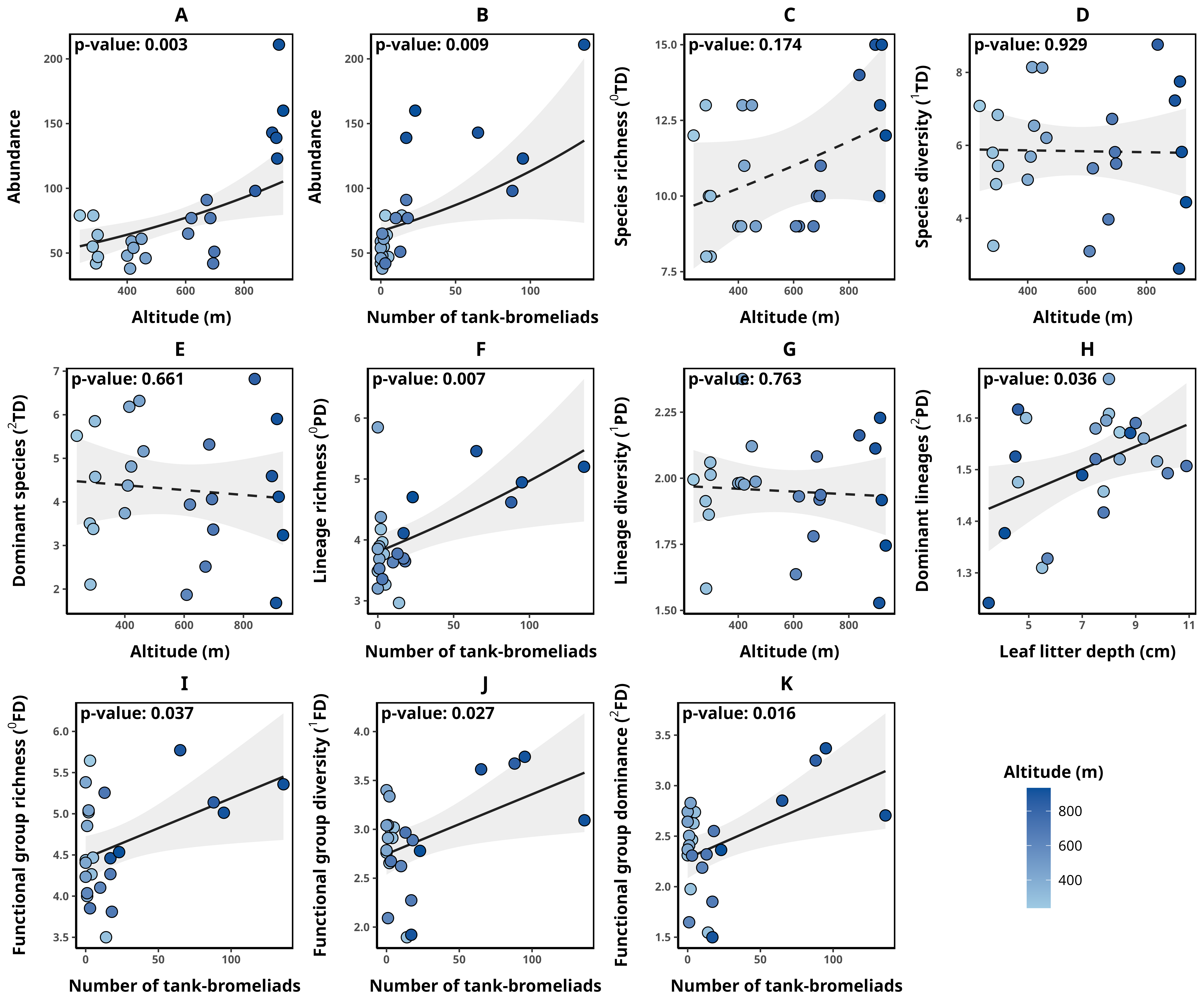

Supplement: Supplemental Information 2 — The R code used to perform the alpha and beta multidimensional diversity analyses, as well as the datasets used in this work. [file peerj-13-19561-s002.zip › R_code/output/diversity_plots/alpha_div.png]

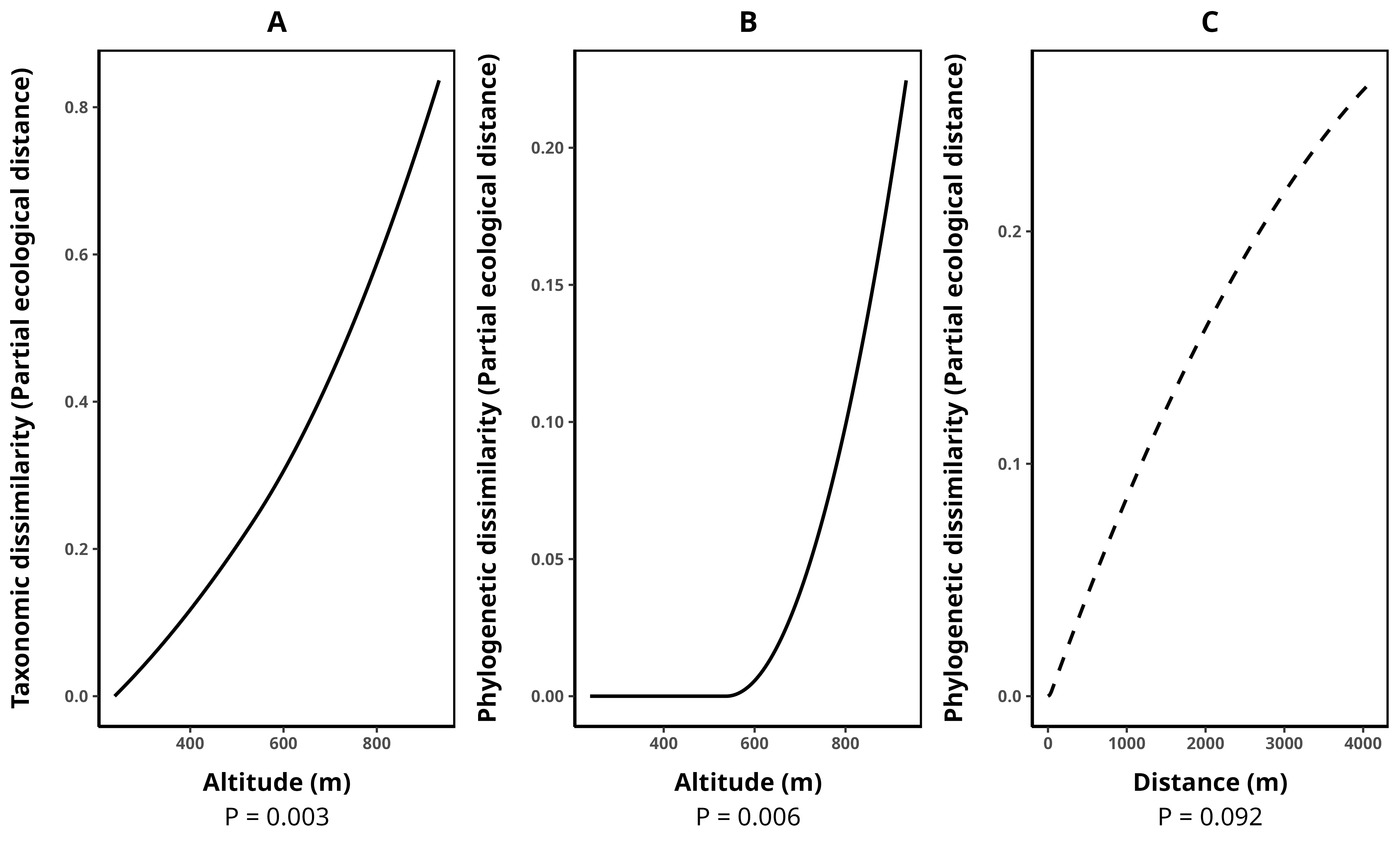

Supplement: Supplemental Information 2 — The R code used to perform the alpha and beta multidimensional diversity analyses, as well as the datasets used in this work. [file peerj-13-19561-s002.zip › R_code/output/diversity_plots/beta.png]

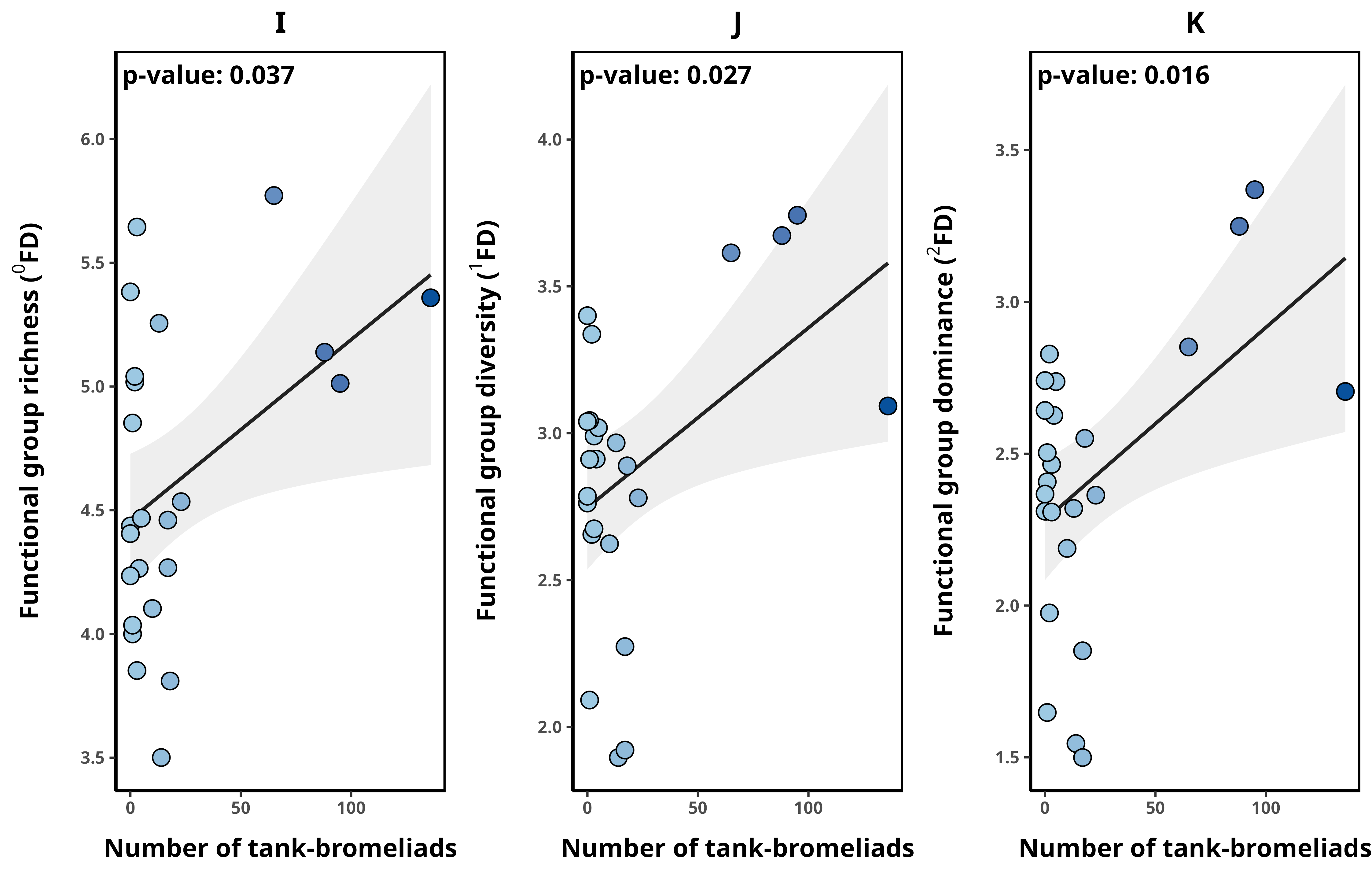

Supplement: Supplemental Information 2 — The R code used to perform the alpha and beta multidimensional diversity analyses, as well as the datasets used in this work. [file peerj-13-19561-s002.zip › R_code/output/diversity_plots/fun_div.png]
